# Hot Deformation and Processing Window Optimization of a 70MnSiCrMo Carbide-Free Bainitic Steel

**DOI:** 10.3390/ma10030318

**Published:** 2017-03-21

**Authors:** Ying Han, Yu Sun, Wei Zhang, Hua Chen

**Affiliations:** 1 Key Laboratory of Advanced Structural Materials, Ministry of Education, Changchun University of Technology, Changchun 130012, China; chenhua@ccut.edu.cn; 2 National Key Laboratory for Precision Hot Processing of Metals, Harbin Institute of Technology, Harbin 150001, China; yusun@hit.edu.cn; 3 State Key Laboratory for Mechanical Behavior of Materials, Xi’an Jiaotong University, Xi’an 710049, China; zhangw_66@163.com

**Keywords:** super bainitic steel, hot compression, flow behavior, DRX, processing map

## Abstract

The hot deformation behavior of a high carbon carbide-free bainitic steel was studied through isothermal compression tests that were performed on a Gleeble-1500D thermal mechanical simulator at temperatures of 1223–1423 K and strain rates of 0.01–5 s^−1^. The flow behavior, constitutive equations, dynamic recrystallization (DRX) characteristics, and processing map were respectively analyzed in detail. It is found that the flow stress increases with increasing the strain rate and decreases with increasing the temperature, and the single-peak DRX can be easily observed at high temperatures and/or low strain rates. The internal relationship between the flow stress and processing parameters was built by the constitutive equations embracing a parameter of Z/A, where the activation energy for hot deformation is 351.539 kJ/mol and the stress exponent is 4.233. In addition, the DRX evolution and the critical conditions for starting DRX were discussed. Then the model of the DRX volume fraction was developed with satisfied predictability. Finally, the processing maps at different strains were constructed according to the dynamic material model. The safety domains and flow instability regions were identified. The best processing parameters of this steel are within the temperature range of 1323–1423 K and strain rate range of 0.06–1 s^−1^.

## 1. Introduction

In recent years, carbide-free bainitic steels have attracted considerable interest because of their unusual combination of roughly 2.5 GPa strength, 700 HV hardness, and 130 MPa·m^1/2^ toughness [1,2,3]. The excellent mechanical properties depend on an ultra-fine alternative microstructure consisting of bainitic ferrite (BF) and carbon-enriched retained austenite (CERA). Compared to the conventional bainitic steels, the smaller size in BF, the absence of carbide coarsening, and the absence of block-shaped retained austenite greatly improve the integrated performance of bainitic steels. As a result, carbide-free bainitic steels are commonly referred to as super bainitic steels, and have been considered as candidate materials for applications in various industrial fields, such as chassis, bearing, side door, railway, and etc. [4,5,6,7,8,9].

However, as we know, the bainitic reaction in the medium-to-high carbon carbide-free bainitic steels is a rather time-consuming process. It may take several days or months to achieve the complete transformation, though the super bainitic microstructure can be obtained easily at low isothermal temperatures [3]. The slow reaction rate for bainitic transformation has undoubtedly become a critical factor that restricts the development of carbide-free bainitic steels in commercial applications. To solve this bottle-neck problem, many efforts have been previously devoted to accelerating the bainitic transformation. The well-established methods are reducing the carbon content in the steel or adding encouraging elements, such as Co and Al [10,11]. However, they make the smelting and subsequent manufacturing of the steel more difficult. Recently, it was confirmed that the prior austenite grain size has a significant effect on the kinetics of the bainitic transformation in super bainitic steels. Refining prior austenite grains can accelerate the bainite reaction because of more nucleation sites that are provided [12]. Moreover, if the grain sizes of austenite are refined to a reasonable range, it will facilitate the increase in growth rate of the bainite sheaves, resulting in a quick complete transformation [13,14]. Hence, the design and control of the grain sizes of austenite are important to increase the bainitic transformation rate to a satisfied time.

Thermo-mechanical processing is an efficacious way to achieve grain refinement and microstructure reconstitution for carbide-free bainitic steels. It is generally known that the microstructures during hot deformation always exhibit various interconnected metallurgical phenomena such as work hardening (WH), dynamic recovery (DRV), and dynamic recrystallization (DRX). These changes in microstructures are sensitive to the processing parameters (e.g., strain rate, deformation temperature, and deformation degree) [15,16,17]. Among which, DRX can eliminate the dislocations caused by WH and ameliorate the hot formability of materials. However, more importantly, the deformed microstructures are replaced by the newly recrystallized equiaxed grains through the DRX. Hence, the desired grain sizes can be obtained by adjusting the DRX process during hot deformation, which will play an important role in accelerating the baintic transformation. In order to effectively control the grain size of prior austenite in carbide-free bainitic steels, it is essential to clarify the hot deformation behavior and microstructural evolution, especially regarding DRX features. Recently, many studies have been carried out on the DRX behavior and optimization of hot processing parameters for various carbon steels and microalloyed steels [18,19,20,21,22,23,24,25,26]. For instance, Zhang et al. [18,19] investigated the hot deformation behavior of 34CrNiMo steel by the DRX kinetic model and processing map in a wide temperature range of 1173–1423 K and a strain rate of 0.002–5 s^−1^. Saadatkia et al. [23] evaluated microstructural changes and hot workability of low and medium carbon steels under strain rate and temperature ranges of 10^−4^–0.1 s^−1^ and 1173–1373 K, respectively, and put emphasis on discussing the effect of the carbon content on the DRX. Rajput et al. [26] studied the DRX characteristics of AISI 1010 steel at temperatures of 1023–1323 K and strain rates of 0.01–20 s^−1^, and the optimal hot processing parameters were proposed. Unfortunately, there is less information available on the hot workability, microstructure evolution, and processing parameter optimization of medium-to-high carbon carbide-free bainitic steels.

High carbon carbide-free bainitic steels (0.6–1.0 wt % C) are paid much attention for their nanostructured bainite and ultra-high strength [27,28,29]. For this type of steel, the ultimate tensile strength of above 2 GPa can be achieved routinely through the isothermal bainitic transformation at low temperatures of 493–523 K [30]. Increasing the heat treatment time during bainitic transformation can obviously enhance the ductility due to the good mechanical stability of the retained austenite [31]. 70MnSiCrMo steel is an internally designed economical high carbon bainitic steel. After the suitable heat treatment, the carbide-free bainitic microstructure can be obtained, which is also supported by the reference [31]. In this steel, the content of Cr is decreased, and the strength and hardenability reductions resulting from the lower Cr content are compensated by suitably increasing the quantity of Mn. The chemical composition modification will also affect the deformation behavior at elevated temperatures. Therefore, the aim of this study focuses on the flow behavior, constitutive equations, and DRX kinetics of 70MnSiCrMo carbide-free bainitic steel by applying the hot compression tests in a wide range of strain rates and temperatures. To reasonably control the microstructures and avoid deformation defects during hot working, the processing maps are derived at different strain levels based on the dynamic material model (DMM). The in-depth research on the effects of variations in the parameters on the processing map and DRX evolution will be a great help for determining the optimal processing parameters in actual production.

## 2. Experimental Procedure

The chemical composition of the carbide-free bainitic steel employed in this study is as follows (wt %): 0.71C, 1.72Si, 1.87Mn, 0.67Cr, 0.25Mo, 0.0068P, 0.0049S, and balanced Fe. The steel was hot rolled square billets and annealed at 1193 K in an air furnace.

**Figure 1:**
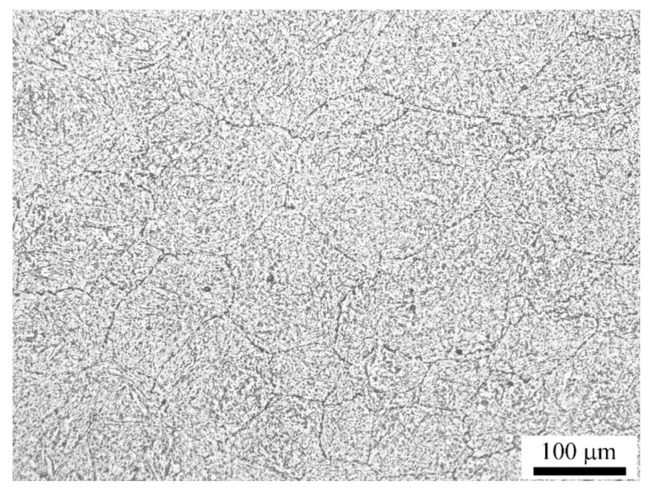
The homogeneous microstructure before hot deformation.

The cylindrical compression specimens of 12 mm in height and 8 mm in diameter were cut from the as-annealed plates and the surface finish reached Ra3.2. The specimens were compression deformed at temperatures ranging from 1223 K to 1423 K in an interval of 50 K and strain rates of 0.01 s^−1^, 0.1 s^−1^, 1 s^−1^, and 5 s^−1^ on a Gleeble-1500D thermo-mechanical simulator. To reduce the friction between the specimen and the die, tantalum foil with a thickness of 0.05 mm has been inserted between them. Each specimen was preheated to 1473 K, held for 180 s for homogenization, and was then cooled to the pre-set deformation temperatures at the cooling rate of 10 K/s in order to simulate the practical industrial process. All the specimens were held for 20 s to eliminate thermal gradients before compression, and then they were deformed to the total true strain of 0.9 and water-quenched immediately. The specimens after deformation were cut along the compression axis and the surfaces were prepared for optical microscopy observation. After normal grinding and polishing processes, the specimens were etched with saturated picric acid. Figure 1 shows the quenched microstructure after holding at 1473 K for 180 s. It can be seen that the microstructure before deformation is homogeneous and the average grain size is about 110 μm.

**Figure 2:**
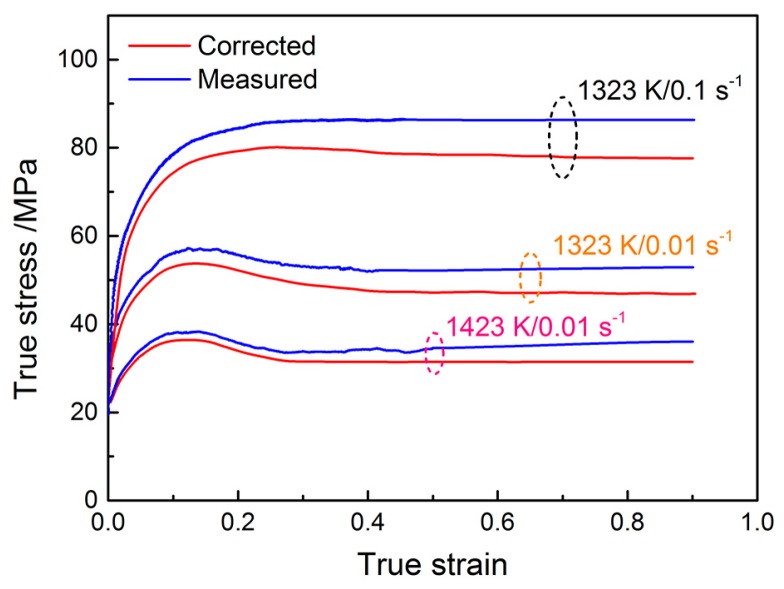
Comparisons between the measured flow curves and friction corrected curves.

The experimental flow stress data were modified in this work because of the friction effect. The effect of friction was accounted for by using the following relation [32]:

(1) $$\bar{\sigma }=\frac{\sigma }{1+\left(2/3\sqrt{3}\right)m\left(r_{0}/h_{0}\right)\exp\left(3\varepsilon /2\right)}$$

where $\bar{\sigma }$, *σ*, *m*, *r*_0_, *h*_0_, and *ε* are the corrected flow stress, measured flow stress, friction factor, initial radius, initial height of specimen, and measured strain, respectively. In this formula, the *m* value is a key parameter for calculating the corrected flow stress data. The detailed calculation process has been described in a previous work by the authors [33]. Figure 2 shows the flow curves before and after friction modification. It is easy to see that the flow stress after modification is much lower than the measured ones, which indicates that the effect of interfacial friction between the specimen and die on the flow behavior is significant. Additionally, if the actual temperature rise is obvious, the flow stress data should be corrected using a linear interpolation between log*σ* versus 1/*T* (*T* is the temperature, K) to reduce the effect of the temperature rise [34]. In this work, the temperature rise for the pre-set deformation temperature can be detected on the flow curve obtained at the strain rate of 5 s^−1^ and temperature of 1223 K, and the temperature increment is only about 5 K. After the modification, the change on the flow curve is not remarkable.

## 3. Results and Discussion

### 3.1. Flow Behavior

**Figure 3:**
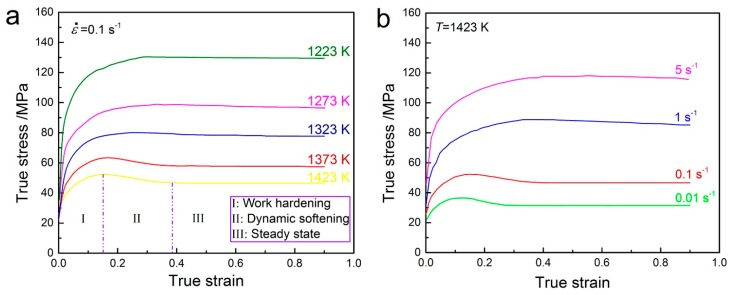
Typical corrected true stress-true strain curves of 70MnSiCrMo carbide-free bainitic steel hot-deformed at (**a**) different temperatures with a strain rate of 0.1 s^−1^ and (**b**) different strain rates with a temperature of 1423 K.

Figure 3 shows the typical corrected true stress-true strain curves of 70MnSiCrMo carbide-free bainitic steel under different working conditions. It is clear that the flow stress decreases with the increase of the deformation temperature and increases with increasing strain rate. The single peak shape can be easily found from the flow curves obtained at high temperatures or low strain rates, which is a typical characteristic of DRX [23,35]. This illustrates that DRX is the dominant softening mechanism under these conditions. Therefore, the variation of flow stress can be divided into three stages marked in Figure 3a. Every stage corresponds to the intrinsic relationship between the flow stress and thermal-dynamic behavior during hot deformation. At the initial stage of deformation (I), the WH plays a leading role due to the rapid proliferation of dislocations and the piling up by obstacles and thus results in the quick increase of the flow stress. With the increase of deformation, the dynamic softening, i.e., DRX caused by dislocation annihilation and rearrangement, becomes predominant and starts to offset or partially offset the effect of WH. At stage (II), the flow stress is decreased continuously. When the multiplication and annihilation of dislocations are in equilibrium, the density of dislocations caused by hot deformation remains constant. Thus the steady flow stress (state III) is achieved. However, there is no distinct peak stress at low temperatures or high strain rates due to the early balance of WH and DRV, implying the softening is controlled by only DRV. As can be seen, the temperature and strain rate have great influence on the flow behavior of 70MnSiCrMo carbide-free bainitic steel at elevated temperatures. In fact, the hot deformation process is thermally activated, which indicates that the high temperature can accelerate the dislocation movement and grain boundary migration, and thus makes the dynamic softening easier to occur. Meanwhile, the low strain rate provides enough time for the nucleation and growth of new DRX grains due to faster dislocation annihilation and more energy accumulation [36].

### 3.2. Z-Parameter and Equations in Flow Softening

For a particular material, the Zener-Hollomon (shortened to *Z*) parameter combines the effects of deformation temperature and strain rate on the deformation characteristics [37]. Moreover, the hyperbolic-sine Arrhenius type constitutive equation proposed by Sellars and Tegart [38] can be commonly applied to construct the connection among flow stress, strain rate, and temperature, embracing the *Z* parameter in a wide range of hot working conditions. They can be expressed as:

(2) $$Z=\dot{\varepsilon }\exp\left(\frac{Q}{RT}\right)$$

(3) $${\left[\sin h\left(\alpha \sigma \right)\right]}^{n}=\frac{Z}{A}=\frac{\dot{\varepsilon }}{A}\exp\left(\frac{Q}{RT}\right)$$

where $\dot{\varepsilon }$ is the strain rate (s^−1^), *Q* is the deformation activation energy (J·mol^−1^), *R* is the gas constant (8.314 mol^−1^·K^−1^), *T* is the deformation temperature (K), *α* is the stress multiplier, *σ* is the flow stress (MPa), *n* is the stress exponent, and *A* is the material constant. Here, the flow stress can be the peak stress (*σ_p_*), steady-state stress (*σ_ss_*), or the one at a particular strain. Commonly, the data of *σ_p_* are likely identified because they are more meaningful in the industrial process. The method to determine *σ_p_* is based on changes of the work hardening rate (*θ*, θ=∂σ/∂ε) [39]. If the value of *θ* is positive, this implies that the work hardening effect is stronger than the dynamic softening. Thus, the *σ_p_* values can be determined at the first occurrence of *θ* = 0 in the single-peak flow curve.

To establish the constitutive relationship of the studied steel, the constants (*α*, *n*, *Q*, and *A*) can be determined by experimental and mathematical analyses. Among them, the value of *α* should be calculated first. According to the level of flow stress in hot deformation, the hyperbolic-sine type Equation (3) can be simplified as:

(4) $$A_{1}\sigma^{n_{1}}=\dot{\varepsilon }\exp\left(\frac{Q}{RT}\right)\;\left(\mathrm{low}\;\mathrm{stress},\alpha \sigma <0.8\right)$$

(5) $$A_{2}\exp\left(\beta \sigma \right)=\dot{\varepsilon }\exp\left(\frac{Q}{RT}\right)\;\left(\mathrm{high}\;\mathrm{stress},\alpha \sigma >1.2\right)$$

where *n*_1_, *β*, *A*_1_, and *A*_2_ are material constants. The ratio between *β* and *n*_1_ represents the *α* (*α* ≈ *β*/*n*_1_). Taking the base-10 logarithm of Equations (4) and (5) gives:

(6) $$\log\sigma =\frac{\log\dot{\varepsilon }}{n_{1}}+\frac{\log^{e}Q}{n_{1}RT}-\frac{\log^{A_{1}}}{n_{1}}$$

(7) $$\sigma =\frac{\log\dot{\varepsilon }}{\log^{e}\beta }+\frac{Q}{\beta RT}-\frac{\log^{A_{2}}}{\log^{e}\beta }$$

**Figure 4:**
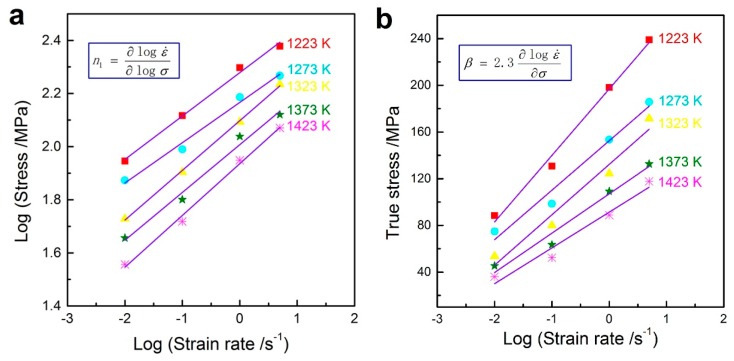
Correlation between flow stress and strain rate at various deformation temperatures: (**a**) log*σ*-log$\dot{\varepsilon }$ and (**b**) *σ*-log$\dot{\varepsilon }$.

The plots of log*σ*-log$\dot{\varepsilon }$ and *σ*-log$\dot{\varepsilon }$ are shown in Figure 4 where the inverse of the slopes of the fitting lines can be used to calculate *n*_1_ and *β*, respectively. It is easily found that the *n*_1_ value ranges from 5.126 to 6.588 and the *β* value ranges from 0.040 to 0.075 MPa^−1^. The average values of *n*_1_ and *β* can be defined as 5.698 and 0.056 MPa^−1^, respectively. Then, *α = β*/*n*_1_ = 0.00976 MPa^−1^.

Subsequently, the obtained *α* value is substituted into Equation (3). *T*aking the base-10 logarithm and re-arranging the equation yields:

(8) $$\log\left[\sin h\left(\alpha \sigma \right)\right]=\frac{\log\dot{\varepsilon }}{n}+\frac{Q}{2.3nRT}-\frac{\log^{A}}{n}$$

By partially differentiating Equation (8), the stress exponent, *n*, can be expressed in terms of

(9) $$n={\frac{\partial \log\dot{\varepsilon }}{\partial \log\left[\sin h\left(\alpha \sigma \right)\right]}|}_{T}$$

**Figure 5:**
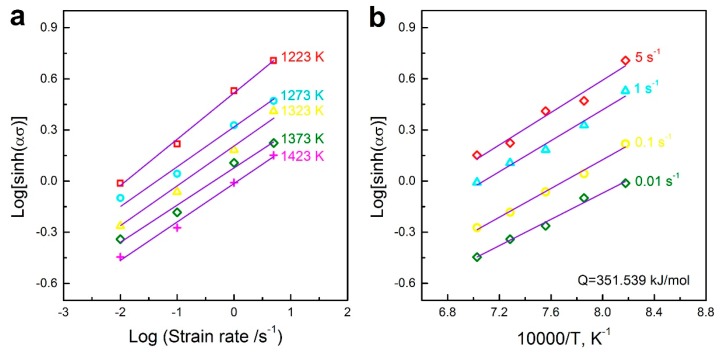
Correlation between (**a**) log[sinh(*ασ*)] vs. log$\dot{\varepsilon }$ at different temperatures and (**b**) log[sinh(*ασ*)] vs. 10,000/*T* at different strain rates.

Figure 5a shows the plots of log[sinh(*ασ*)] vs. log$\dot{\varepsilon }$ at different temperatures. It is seen that the fitting lines match well with the experimental data, and the parallel relationship of these straight lines indicates that the selected *α* value is appropriate in the present working condition. By averaging the inverse of the slopes of the fitting lines, the value of *n* is determined as 4.233.

Similarly, by partially differentiating Equation (8) again, the deformation activation energy, *Q*, which is an important physical parameter indicating the hot workability of materials, can be defined as

(10) $$Q=2.3nR{\frac{\partial \log\left[\sin h\left(\alpha \sigma \right)\right]}{\partial \left(1/T\right)}|}_{\dot{\varepsilon }}$$

Based on the plots of log[sinh(*ασ*)] against 10,000/*T* at different strain rates, as shown in Figure 5b, the values of *Q*/(2.3*nR*) are acquired from the slopes of the fitting lines, and thus, the *Q* value corresponds to 351.539 kJ/mol.

Taking the base-10 logarithm on both sides of Equation (3) yields a new formula

**Figure 6:**
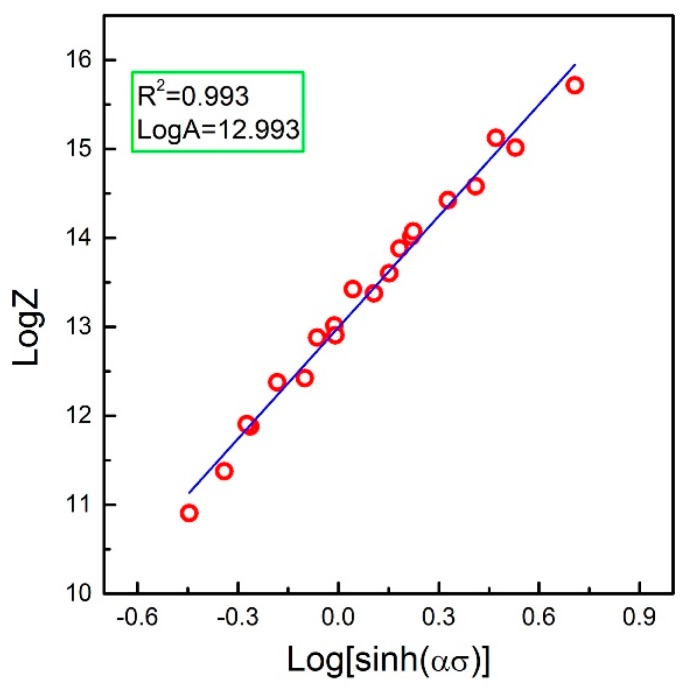
Correlation between log[sinh(*ασ*)] vs. log*Z* under different working conditions.

(11) logZ=logA+nlog[sinh(ασ)]

where the value of *A* can be easily obtained. By inputting the peak stress, strain rate, temperature, and material constants (*α*, *n*, and *Q*) into Equation (11), the relationship between ln[sinh(*ασ*)] and log*Z* can be drawn, as shown in Figure 6. The flow stress increases with increasing the *Z* parameter. Meanwhile, a good linear fit for the data plots is observed, and the value of log*A* can be determined by the intercept. Hence, the parameter of *A* is defined as 1.371 × 10^11^.

Re-writing Equation (11), the peak stress can be expressed as a function of (*Z*/*A*). This equation is as follows:

(12) σ=1αln[(ZA)1n+(ZA)2n+1]

The peak stress of 70MnSiCrMo carbide-free bainitic steel during hot compression can therefore be presented as:

(13) $$\sigma =102.459\ln\left[{\left(\frac{Z}{1.371\times 10^{11}}\right)}^{0.236}+\sqrt{{\left(\frac{Z}{1.371\times 10^{11}}\right)}^{0.472}+1}\right]$$

where the *Z* parameter is expressed as $Z=\dot{\varepsilon }\exp\left(\frac{351539}{RT}\right)$.

**Figure 7:**
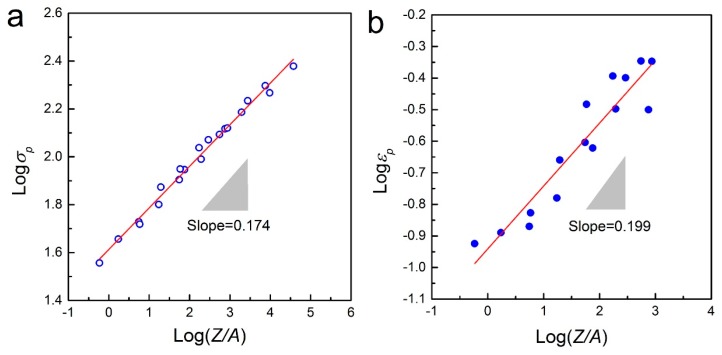
Correlations between (**a**) log*σ*_p_ vs. log(*Z*/*A*) and (**b**) log*ε*_p_ vs. log(*Z*/*A*).

For simple representation, the peak stress and the corresponding strain, *ε*_p_, can be expressed as a linear relation with log(*Z*/*A*) [37,40]. Figure 7 shows the plots of log*σ*_p_ vs. log(*Z*/*A*) and log*ε*_p_ vs. log(*Z*/*A*) respectively. By regression analysis of the data points, the following equations are obtained:

(14) $$\sigma_{p}=41.02{\left(\frac{Z}{A}\right)}^{0.174}$$

(15) $$\varepsilon_{p}=0.115{\left(\frac{Z}{A}\right)}^{0.199}$$

**Figure 8:**
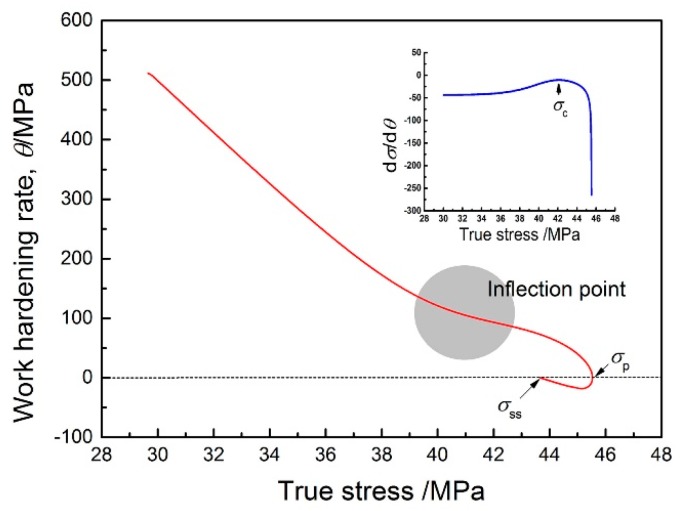
An example for identifying the values of *σ*_ss_ and *σ*_c_ through plotting *θ* vs. *σ* and *dθ*/*dσ* vs. *σ* (1373 K/0.01 s^−1^).

**Figure 9:**
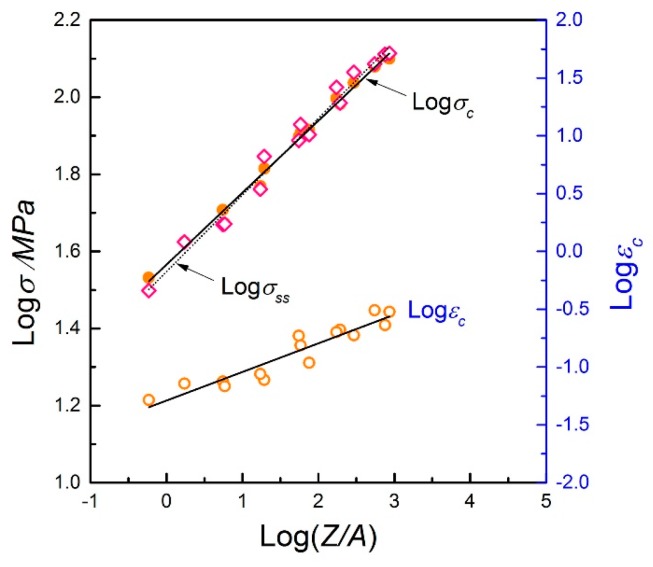
Relationships between the dimensionless parameter, *Z*/*A*, with *σ*_c_, *σ*_ss_, and *ε*_c_, respectively.

Besides the peak values, the critical stress (*σ*_c_), critical strain (*ε*_c_), and steady stress (*σ*_ss_) obtained from the flow curves are also quite important characteristic parameters to describe the softening behavior. The values of *σ*_c_ and *ε*_c_ represent the critical condition for the initiation of DRX, while *σ*_ss_ is related to the microstructural mechanism during hot deformation. In this work, the values of *σ*_ss_ are identified from the relationship curves of *θ* vs. *σ* at different deformation conditions*.* Due to the difficulty in reading the value of *σ*_c_ directly, the method proposed by Poliak and Jonas [41] is employed, which involves plotting *dθ*/*dσ* vs. *σ.* Figure 8 gives an example to evaluate the values of *σ*_c_ and *σ*_ss_. The value of *ε*_c_ can be obtained by mapping the *σ*_c_ value back into the flow curve. These characteristic parameters can also be represented as a function of log(*Z*/*A*), as shown in Figure 9, which successfully builds the relationship between the flow behavior and working variables. They are expressed as

(16) $$\sigma_{c}=36.728{\left(\frac{Z}{A}\right)}^{0.187}$$

(17) $$\varepsilon_{c}=0.0513{\left(\frac{Z}{A}\right)}^{0.248}$$

(18) $$\sigma_{ss}=35.237{\left(\frac{Z}{A}\right)}^{0.199}$$

It is seen that the DRX can easily take place at high temperatures and low strain rates, where a larger flow steady state would be obtained. Additionally, the relationship in the form of *ε*_c_ ≈ 0.779*ε*_p_ can be acquired through the regression analysis, which intuitively reveals when the DRX starts in the 70MnSiCrMo carbide-free bainitic steel during hot deformation.

### 3.3. DRX Kinetic Model

The evolution of the DRX process in the metal and alloys strongly depends on the changes of movement and density of dislocation during hot deformation. When the dislocations pile up ahead of grain boundaries and deformation bands to a critical density, DRX starts to occur. This process is generally affected by the working parameters. To describe the DRX kinetics during hot deformation, a mathematical model based on the Avrami equation is employed [19,21,23]:

(19) $$X_{\mathrm{DRX}}=1-\exp\left[-k{\left(\frac{\varepsilon -\varepsilon_{c}}{\varepsilon_{p}}\right)}^{n}\right]$$

where *X*_DRX_ represents the volume fraction of DRX, and *k* and *n* are material constants. In fact, the determination of *X*_DRX_ is rather difficult by metallographic measurements due to the large workload and high error. Hence, there is a common agreement for obtaining *X*_DRX_ by the variation of flow stress with the strain at different conditions [19,37,42,43]. The relationship between *X*_DRX_ and flow stress can be expressed by the following equation:

(20) $$X_{\mathrm{DRX}}=\frac{\sigma_{sat}-\sigma }{\sigma_{sat}-\sigma_{ss}}$$

where *σ_s_*_at_ is the saturated stress. The value of *σ_s_*_at_ can be easily calculated, and the detailed method was reported earlier in Reference [44].

Combining with Equations (19) and (20) gives:

(21) $$\frac{\sigma_{sat}-\sigma }{\sigma_{sat}-\sigma_{ss}}=1-\exp\left[-k{\left(\frac{\varepsilon -\varepsilon_{c}}{\varepsilon_{p}}\right)}^{n}\right]$$

Taking the logarithm of both sides of Equation (21) yields:

(22) $$\ln\left[-\ln\left(1-X_{\mathrm{DRX}}\right)\right]=\ln k+n\ln\left(\frac{\varepsilon -\varepsilon_{c}}{\varepsilon_{p}}\right)$$

**Figure 10:**
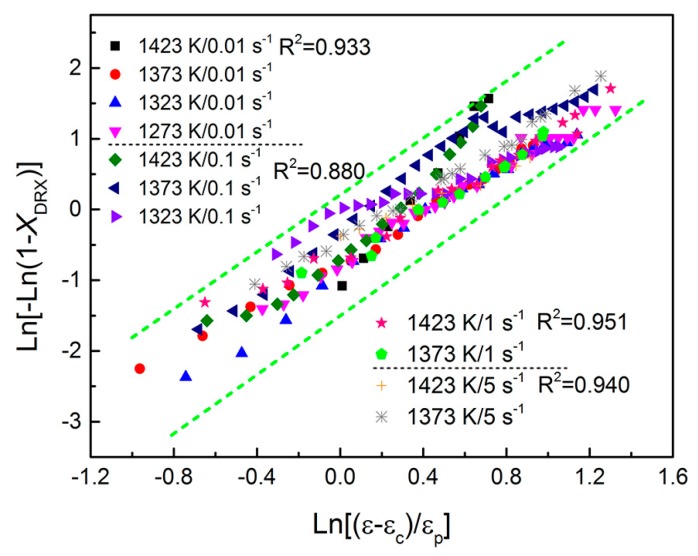
Relationship between ln[−ln(1 − *X*_DRX_)] and ln[(*ε* − *ε*_c_)/*ε*_p_] under different working conditions.

Substituting the obtained values of *ε*_c_, *ε*_p_, *σ_s_*_at_, and *σ_s_*_s_ at various conditions into Equation (22), the plot of ln[−ln(1 − *X*_DRX_)] and ln[(*ε* − *ε*_c_)/*ε*_p_] can be drawn, as shown in Figure 10. The linear regression at different levels of strain rate is conducted and the good fit for the data points can be observed. The *n* values at strain rates of 0.01 s^−1^, 0.1 s^−1^, 1 s^−1^, and 5 s^−1^ are 1.800, 1.711, 1.764, and 1.649, respectively. And the ln*k* values at strain rates of 0.01 s^−1^, 0.1 s^−1^, 1 s^−1^, and 5 s^−1^ are −0.754, −0.361, −0.670, and −0.495, respectively. Then the final values of *k* and *n* are determined by averaging the ones at different strain rates, and they are 0.566 and 1.731, respectively. The corresponding standard deviations are 0.176 and 0.0658. Therefore the model of the DRX volume fraction for 70MnSiCrMo carbide-free bainitic steel can be expressed as follows:

(23) $$X_{\mathrm{DRX}}=1-\exp\left[-0.566{\left(\frac{\varepsilon -\varepsilon_{c}}{\varepsilon_{p}}\right)}^{1.731}\right]$$

**Figure 11:**
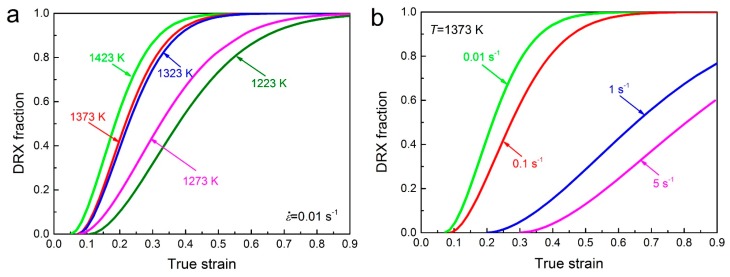
Volume fractions of dynamic recrystallization (DRX) obtained at (**a**) different temperatures with the strain rate of 0.01 s^−1^ and (**b**) different strain rates with the temperature of 1373 K.

Based on Equation (23), the DRX kinetic curves of 70MnSiCrMo carbide-free bainitic steel at different temperatures and strain rates are shown in Figure 11. It is obvious that the DRX volume fraction increases toward 1 (100% DRX) with increasing strain in terms of the *S*-shape. Increasing temperature or decreasing strain rate can speed up the DRX process because the migration rate of dislocations and grain boundaries becomes higher, and thus results in a remarkable increment in the amount of DRX volume fraction at the same strain.

**Figure 12:**
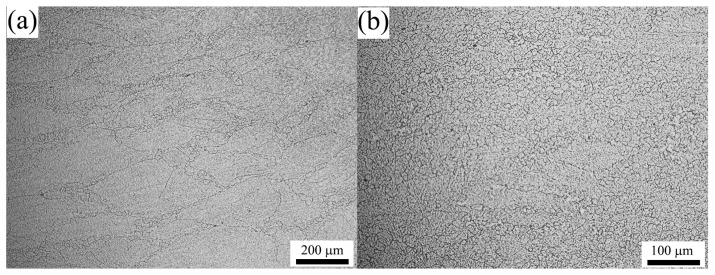
Typical deformed microstructures of 70MnSiCrMo carbide-free bainitic steel obtained at 1373 K and 0.1 s^−1^ with low strains of (**a**) 0.162 and (**b**) 0.357.

**Figure 13:**
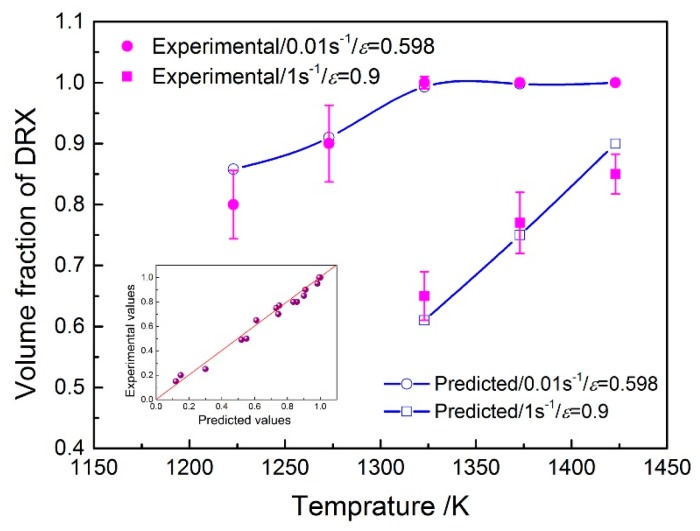
Comparisons between the predicted and experimental DRX volume fractions at strain rates of 0.01 s^−1^ and 1 s^−1^. The inset figure is the correlation between the predicted and experimental ones for more working conditions.

To evaluate the precision of the developed DRX model, the metallographic observation is performed under typical conditions and the microstructures obtained at 1373 K and 0.1 s^−1^ with low strains are shown in Figure 12. It can be seen that the DRX volume fraction indeed increases as the strain increases, and the values at strains of 0.162 and 0.357 are about 12% and 65%, respectively. More comparisons between the experimental and the predicted data have been carried out, as shown in Figure 13. As is seen, the predicted DRX volume fractions (the plots) agree well with the experimental ones (the points). The calculated value of *R* is 0.985 (Figure 13 inset), which confirms that the model can be applied to predict the volume fraction of DRX for 70MnSiCrMo carbide-free bainitic steel in a hot working process with good precision.

### 3.4. Processing Map and Microstructures

The processing map proposed by Prasad and his co-workers is a useful method to identify the optimal working parameters and forecast the microstructure and properties of products [45]. In recent years, the processing map has been successfully applied in a wide range of metallic materials, such as magnesium, aluminum, titanium, Ni-based alloys, and steels [36,39,46,47,48,49]. The basic principle of the processing map is the dynamic materials model (DMM) where the work piece during hot deformation is considered to be a dissipater of the instantaneous input power and the power (*P*) can be separated into two complementary functions, as shown in Equation (24).

(24) $$P=\sigma \dot{\varepsilon }=G+J=\int_{0}^{\dot{\varepsilon }}\sigma d\dot{\varepsilon }+\int_{0}^{\sigma }\dot{\varepsilon }d\sigma$$

where *G* represents the viscoplastic heat caused by plastic deformation and *J* is related to the power dissipation through the microstructure changes (i.e., DRV, DRX, cavity formation, and phase transformation). Commonly, a dimensionless parameter (*η*), named the power dissipation efficiency, is used to reflect the power dissipation capacity for microstructural changes. It is given by the following equation:

(25) $$\eta =\frac{2m}{m+1}$$

where *m* is the strain rate sensitivity which is defined as $\left(\partial \log\sigma \right)/\left(\partial \log\dot{\varepsilon }\right)$.

The variation of the power dissipation efficiency with the strain rate and deformation temperature constitutes a power dissipation map. The power consumption at various strains is different and every domain in the power dissipation map directly corresponds to a specific microstructure evolution mechanism. In general, the high value of *η* indicates the occurrence of DRX during hot deformation which thus leads to the reconstruction of the original microstructure. However, the phenomenon of flow instability may occur in the hot deformation, which can result in deformation defects such as flow localization, deformation bands, and cracks. Hence, the flow instability should be avoided and the precise prediction of deformation defects is very necessary. Here, the extremum principles of irreversible thermodynamics as applied to continuum mechanics of large plastic flow are employed to define whether flow instability has occurred [50]. The instability criterion is derived by

(26) $$\xi \left(\dot{\varepsilon }\right)=\frac{\partial \log\left(\frac{m}{m+1}\right)}{\partial \log\dot{\varepsilon }}+m\leq 0$$

where $\xi \left(\dot{\varepsilon }\right)$ is a dimensionless instability parameter. Similarly, the variation of $\xi \left(\dot{\varepsilon }\right)$ with the strain rate and deformation temperature constructs the instability map. If the values of $\xi \left(\dot{\varepsilon }\right)$ are negative, the flow instability would occur and the corresponding domains are unsafe for hot working. By contrast, the positive instability parameter indicates that the material can be deformed safely. The processing map can be obtained through superimposing the power dissipation map on the instability map.

**Figure 14:**
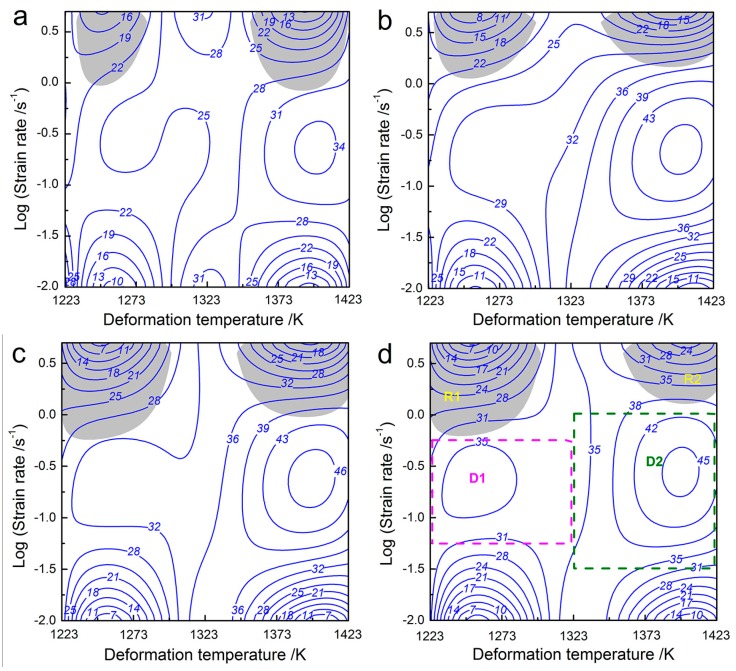
Processing maps of 70MnSiCrMo carbide-free bainitic steel at various strains: (**a**) 0.162; (**b**) 0.357; (**c**) 0.598; (**d**) 0.9. The square domains (D1 and D2) possess a high efficiency of power dissipation and are considered to be the suitable processing windows, while the gray regimes (R1 and R2) indicate that it is unsafe for processing.

Figure 14 shows the processing maps of 70MnSiCrMo carbide-free bainitic steel at different strains from 0.162 to 0.9 which correspond to the compression reductions of 15%–60%. In these maps, the contour number denotes the efficiency of power dissipation in percent while the gray regimes with negative $\xi \left(\dot{\varepsilon }\right)$ are termed unsafe for processing. It is seen that the changing trends of the power dissipation efficiency with temperature and strain rate at the strain range of 0.162–0.9 are similar. In most domains, the value of the power dissipation efficiency increases with increasing the true strain, but it decreases marginally when the strain is greater than 0.598. As shown in Figure 14d, when the flow behavior reaches the stable stage (or large strain), it exhibits two domains with peak efficiency in the processing map. The first domain is in the temperature range of 1223–1323 K and strain rate range of 0.05–0.56 s^−1^ and the peak power dissipation efficiency is 35%. Another domain is located in the temperature window of 1323–1423 K and strain rate range of 0.03–1 s^−1^, where the efficiency of power dissipation is more than 42%. Generally, the high efficiency values of 30%–50% are associated with DRX which is considered to be the optimum microstructure for hot working because it will keep the stress and WH rate low while maintaining high ductility [16]. The larger safety domains based on the peak efficiency of power dissipation also facilitate the design and control of DRX grain sizes. In addition, the processing maps also predict the flow instabilities for different strain levels. It can be seen that there are two unstable regimes located at high strain rates that are higher than 1 s^−1^. With the true strain increasing from 0.162 to 0.9, the first unstable regime occurred at the low temperature range of 1233–1280 K and strain rate range of 1–5 s^−1^ and enlarges slightly, while the secondary unstable regime located around the high temperature range of 1353–1423 K and strain rate range of 0.7–5 s^−1^ has shrunk. Furthermore, these unstable regimes have a small efficiency of power dissipation, which means that the dissipative energy for the microstructural change during hot deformation is low. Consequently, the processing windows corresponding to the identified instability regimes are not favorable for the hot working of 70MnSiCrMo carbide-free bainitic steel.

**Figure 15:**
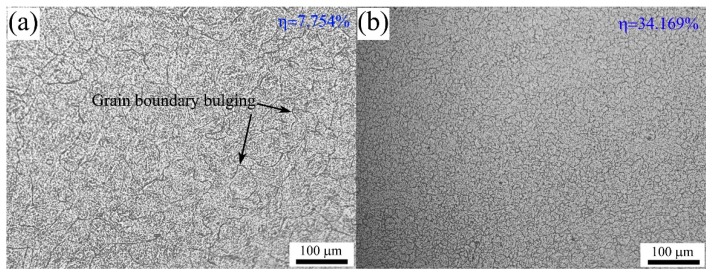

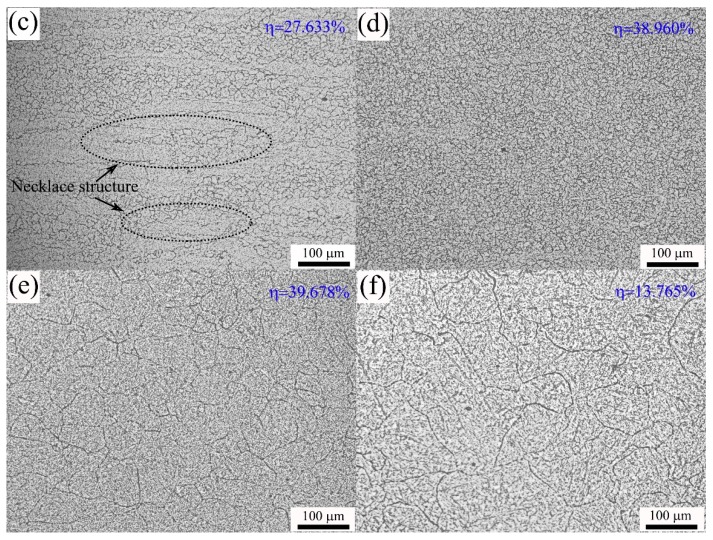
Typical microstructures of 70MnSiCrMo carbide-free bainitic steel after hot deforming at different temperatures and strain rates: (**a**) 1273 K/0.01 s^−1^; (**b**) 1273 K/0.1 s^−1^; (**c**) 1273 K/1 s^−1^; (**d**) 1423 K/1 s^−1^; (**e**) 1423 K/0.1 s^−1^; (**f**) 1423 K/0.01 s^−1^.

**Figure 16:**
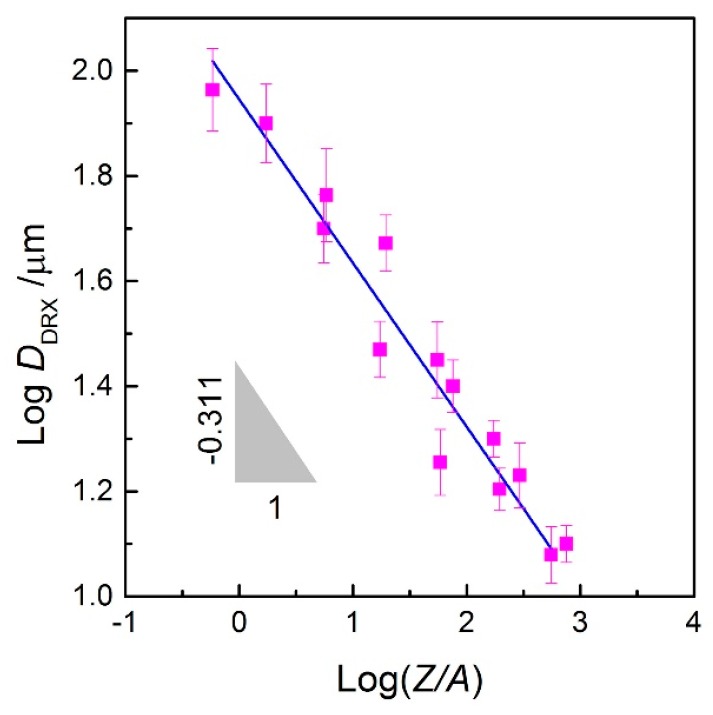
Correlation between log*D*_DRX_ vs. log(*Z*/*A*) at various deformation conditions.

Figure 15 shows the typical microstructures of 70MnSiCrMo carbide-free bainitic steel after hot deforming at different temperatures and strain rates. Figure 15a presents the microstructure obtained at the condition of 1273 K/0.01 s^−1^. In this figure, the uniform DRX grains are visible and the average grain size is about 47 μm, illustrating that the complete DRX microstructure can be developed at a low enough strain rate, though, at a low deformation temperature. It is particular noted that most grains exhibit irregular shapes characterized with wavy or serrated grain boundaries. Therefore, it is deduced that the growth of DRX grains at this state is not terminated. Meanwhile, the corresponding power dissipation efficiency is only 7.754%, which further confirms that the present processing parameters are not optimal for hot working. The occurrence of wavy or serrated grain boundaries indicates that the grain boundary bulging is the nucleation mechanism of DRX [51,52]. Figure 15b shows the microstructure of the steel deformed at a strain rate of 0.1 s^−1^ and a temperature of 1273 K with a power dissipation efficiency of 34.169%. It can be seen that the original coarse grains have evenly transformed into the newly formed equiaxed DRX grains with a small size of 16 μm, indicating that high energy dissipation in this condition is used for the nucleation of DRX. Compared with that in Figure 15a, the degree of DRX is slightly decreased but the grain size is much finer. This is because the high strain rate provides more nucleation sites for DRX and therefore promotes the DRX formation. However, when the strain rate is increased to 1 s^−1^, as shown in Figure 15c, the microstructure represents some necklace structures which are featured by many newly fine grains along original grain boundaries, and thus the primary grains are gradually decorated by the DRX grains but not completed. The degree of DRX is obviously decreased. Moreover, uneven deformation has occasionally occurred at this condition and therefore it is located in the instable regime. Hence, the DRX behavior is very sensitive to the strain rate and the excessive strain rate goes against the growth of DRX grains. With increasing the temperature to 1423 K, coarser DRX microstructures are obtained, as shown in Figure 15d,e, and the mean grain sizes are about 18 μm and 58 μm, respectively. The higher the deformation temperature, the larger the driving force for DRX nucleation, the faster the migration rate of the strain-induced grain boundary, and the greater the DRX degree. Figure 15f shows the microstructure of the steel deformed at a strain rate of 0.01 s^−1^ and temperature of 1473 K where the power dissipation efficiency is 13.765%. A significant number of irregular DRX grains are found and the grain size is increased to 92 μm. This is because the low strain rate provides enough time for new grains to grow at high temperatures. To reveal the variations of the DRX grain size with deformation conditions, the correlation between the grain size (*D*_DRX_) and the dimensionless parameter, *Z*/*A* is developed, as shown in Figure 16. As seen, the relationship between them can be analyzed by linear regression and thus expressed by Equation (27). Using this equation, one can control and predict the grain size of 70MnSiCrMo carbide-free bainitic steel after hot deformation.

(27) $$D_{\mathrm{DRX}}=88.308{\left(\frac{Z}{A}\right)}^{-0.311}$$

**Figure 17:**
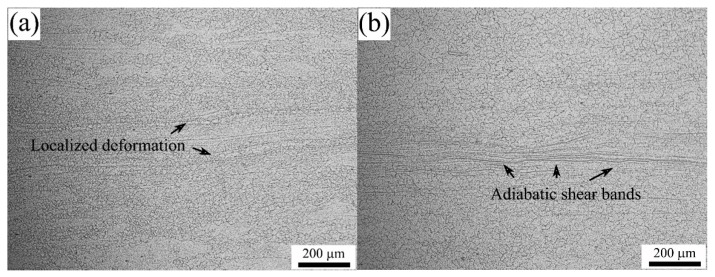
Typical instable microstructures of 70MnSiCrMo carbide-free bainitic steel: (**a**) 1273 K/5 s^−1^; (**b**) 1423 K/5 s^−1^.

The typical microstructures in the instability regimes are also observed, as shown in Figure 17a,b. From Figure 17a, the microstructure corresponding to the condition of 1273 K/5 s^−1^ exhibits features of mixed grains caused by intensely centralized deformation during the hot compression. This is a typical form of localized flow which commonly causes large internal stress and facilitates crack initiation and propagation by further deformation [53], and thus is harmful to acquiring good mechanical properties. Moreover, the low power dissipation efficiency in this region also suggests that most of the plastic power input converts to heat and dissipates in the form of a temperature rise in the steel [36,46]. The microstructure of the steel deformed at a strain rate of 5 s^−1^ and temperature of 1473 K is shown in Figure 17b. It can be seen that the adiabatic shear bands consisting of numerous fine grains have been formed, which may be associated to cracks and cavity [54]. At high strain rates, the heat generated from the hot deformation conducted at high temperatures cannot escape timely and then the internal temperature rise is unavoidable. The local flow softening and local DRX are mainly responsible for these shear deformation bands. Hence, the identified instable regimes should be kept away during practical processing.

Based on this analysis, it is summarized that the DRX behavior and equiaxed grain size are sensitive to the deformation temperature and strain rate. The hot deformation at an intermediate strain rate is beneficial for obtaining a DRX microstructure with fine equiaxed grains. The high strain rate (above 1 s^−1^) can increase the sensitivity of 70MnSiCrMo carbide-free bainitic steel to deformation instability. On the other hand, the higher the deformation temperature, the easier the DRX occurrence, and the faster the migration rate of the grain boundaries. This leads to the formation of a coarser DRX microstructure. Therefore, the deformation conditions of 1223–1323 K/0.05–0.56 s^−1^ and 1323–1423 K/0.03–1 s^−1^ can be used to control the grain size of 70MnSiCrMo carbide-free bainitic steel, which provides a reference for accelerating the subsequent isothermal bainitic reaction. However, if we want to obtain a reasonable DRX microstructure and good hot workability, the optimal processing parameters are the temperature range from 1323–1423 K and strain rate range from 0.06–1 s^−1^.

## 4. Conclusions

The hot compression behavior of 70CrMnSiMo carbide-free bainitic steel has been investigated in the temperature range from 1223–1423 K and strain rate range from 0.01–5 s^−1^. The following conclusions are drawn from this study:

(1) The flow stress is strongly dependent on the deformation temperature and strain rate during hot compression, and it increases with increasing the strain rate, and decreases with the increase in temperature. The single peak stress can be easily found on the flow curves obtained at high temperatures and/or low strain rates, which implies that the DRX is responsible for the dynamic softening under these conditions.
(2) The constitutive equation embraced by the parameter of *Z*/*A* is developed, where the hot deformation activation energy is 351.539 kJ/mol. In addition, the critical conditions for the occurrence of DRX are identified throughout the hot working range and the ratio between the critical strain and peak strain is about 0.779.
(3) The process of DRX can be accelerated remarkably by increasing the deformation temperature or decreasing the strain rate. The model of the DRX volume fraction during hot compression is developed based on the Avrami equation as follows: $X_{\mathrm{DRX}}=1-\exp\left[-0.566{\left(\frac{\varepsilon -\varepsilon_{c}}{\varepsilon_{p}}\right)}^{1.731}\right]$. The comparative evaluation indicates that the model has good capability to describe and predict the kinetic behavior of DRX.
(4) The processing map at different true strains was established based on DMM. The strain has obvious impacts on the efficiency of the power dissipation and the instability parameter. To obtain a reasonable DRX and good hot workability, the optimum hot working parameters are the temperature range from 1323–1423 K and strain rate range from 0.06–1 s^−1^ with a peak power dissipation efficiency of 45%. The instability regions caused by localized deformation and adiabatic shear are located at high strain rates, which should be avoided during hot processing.
